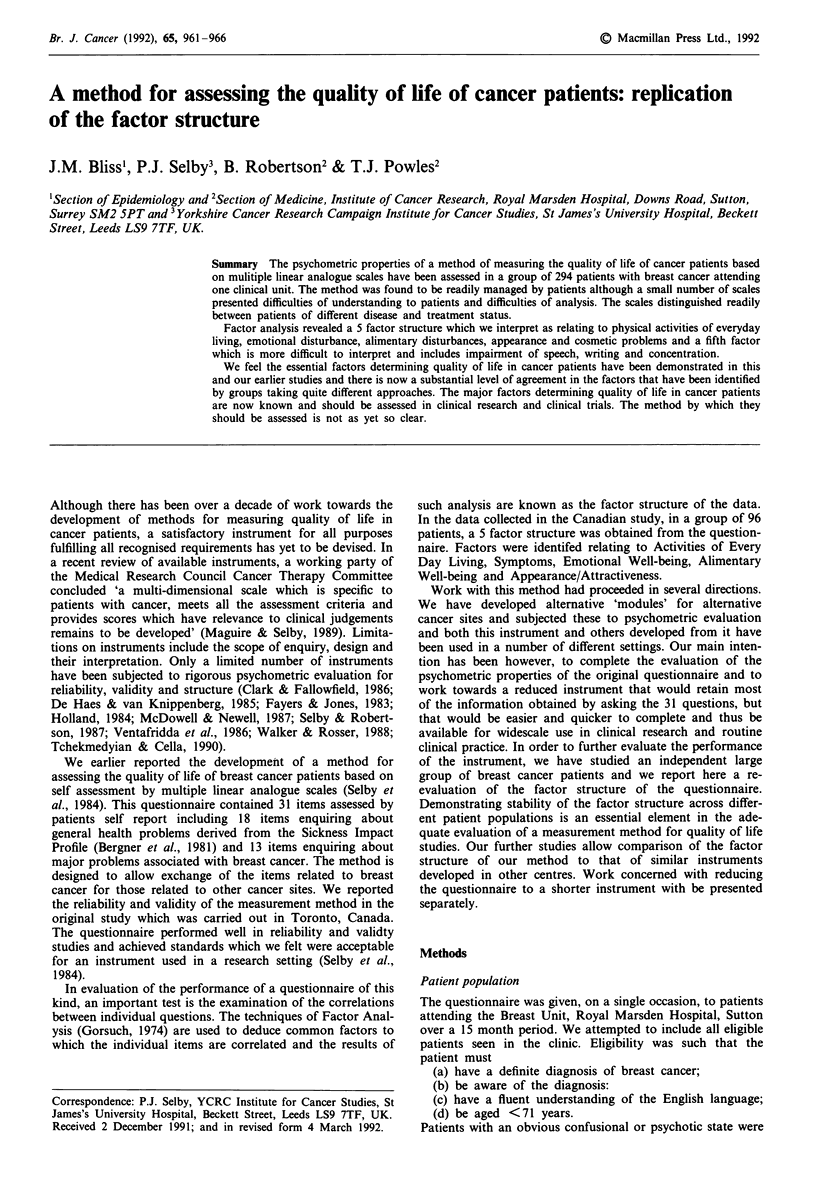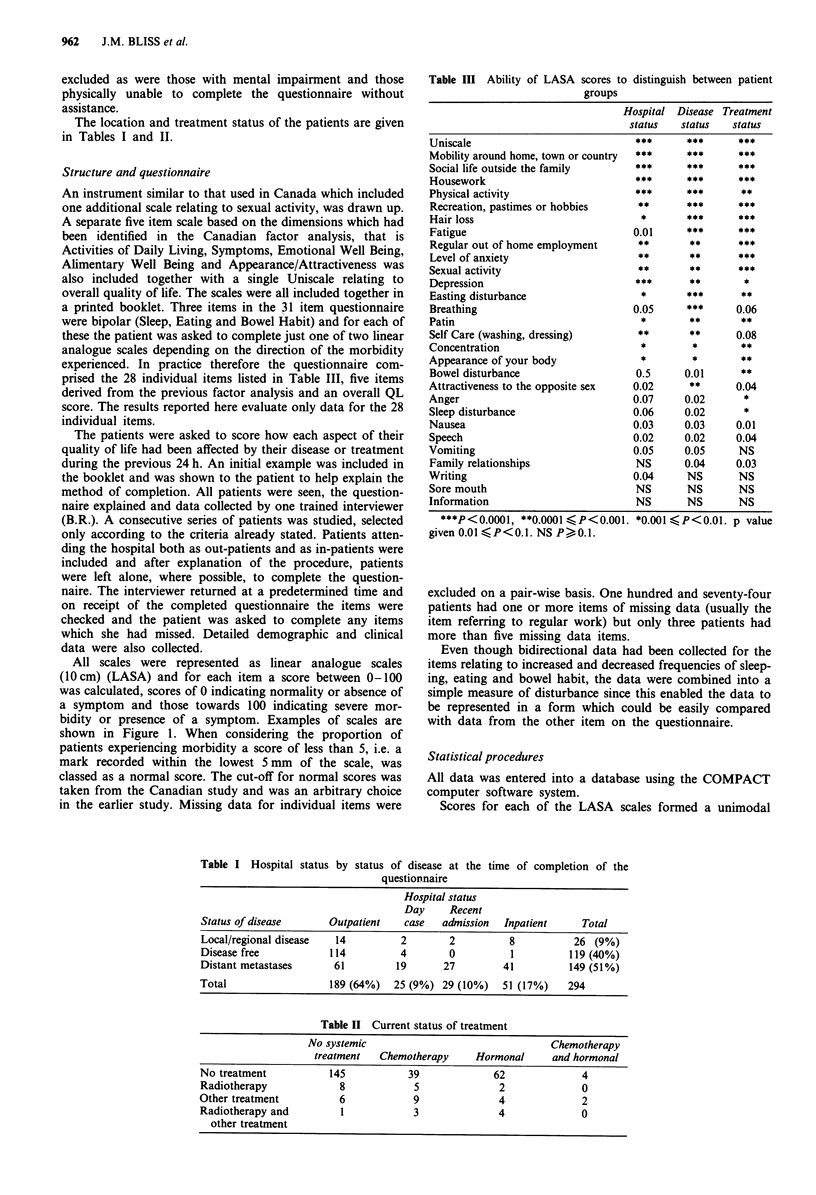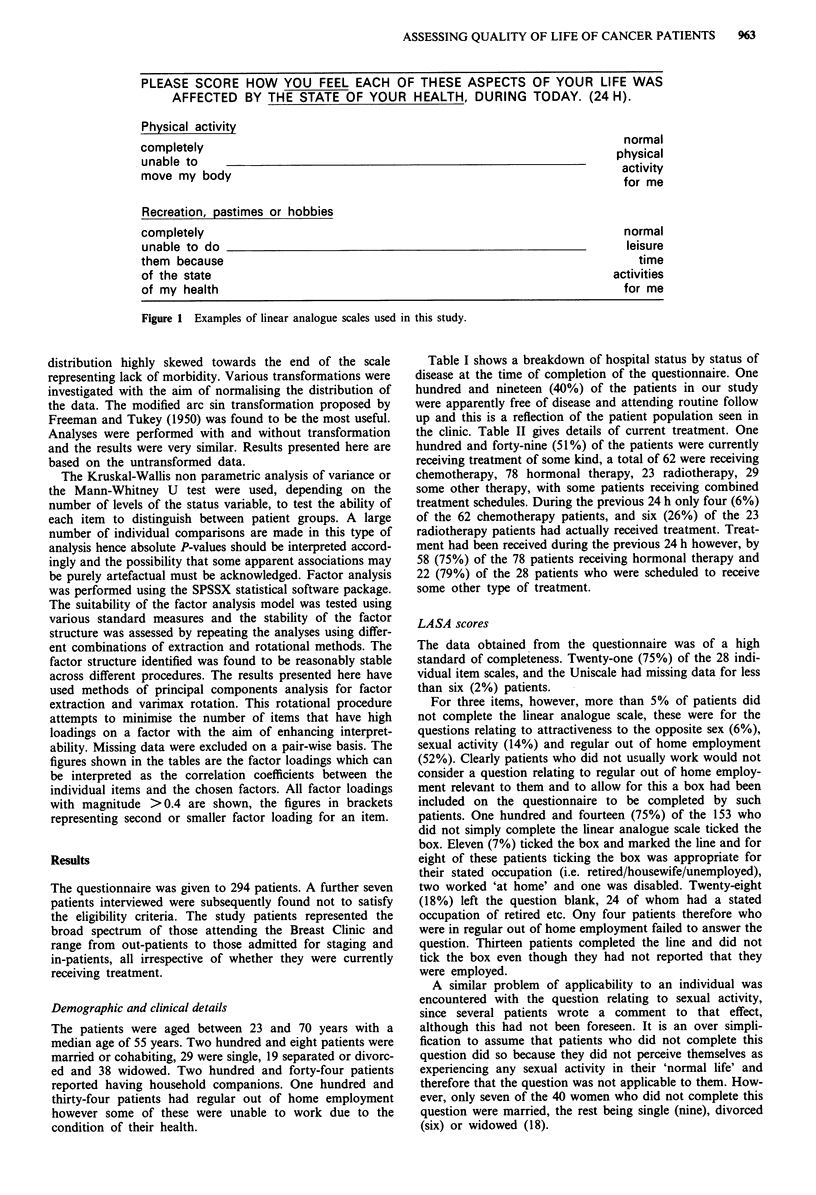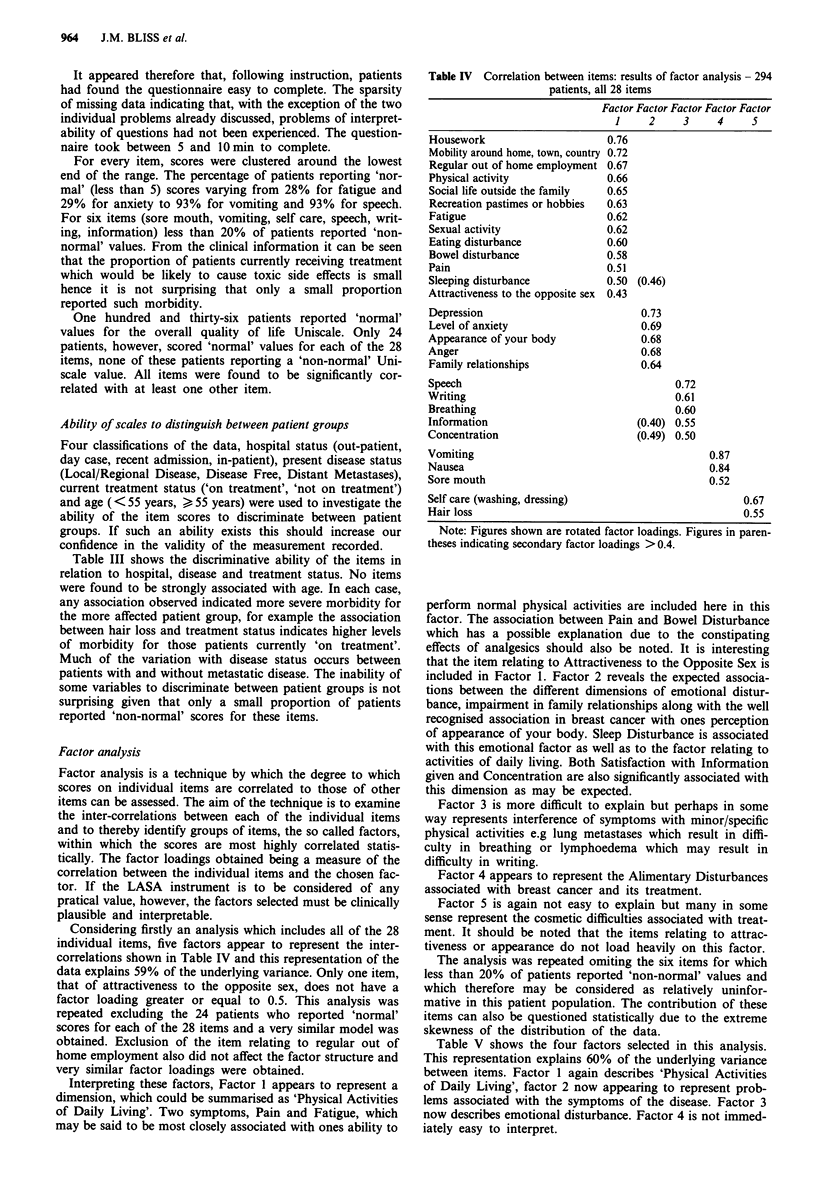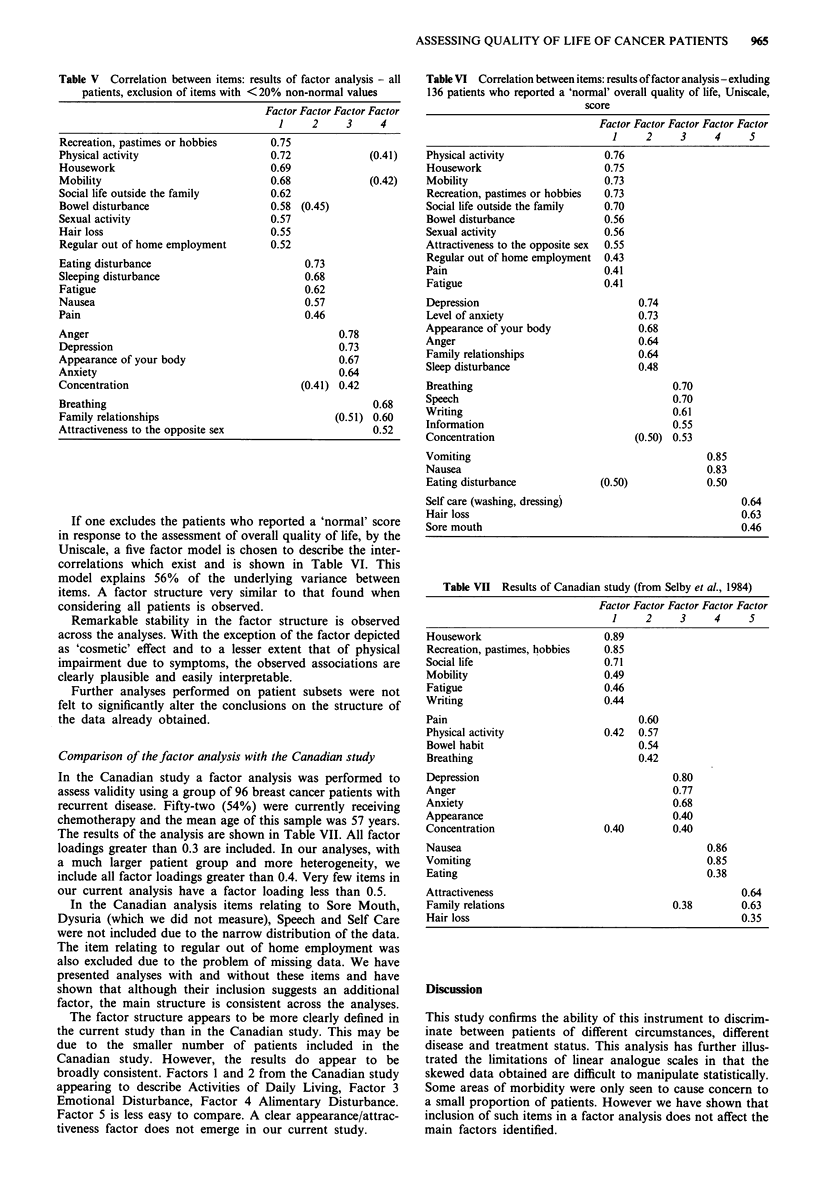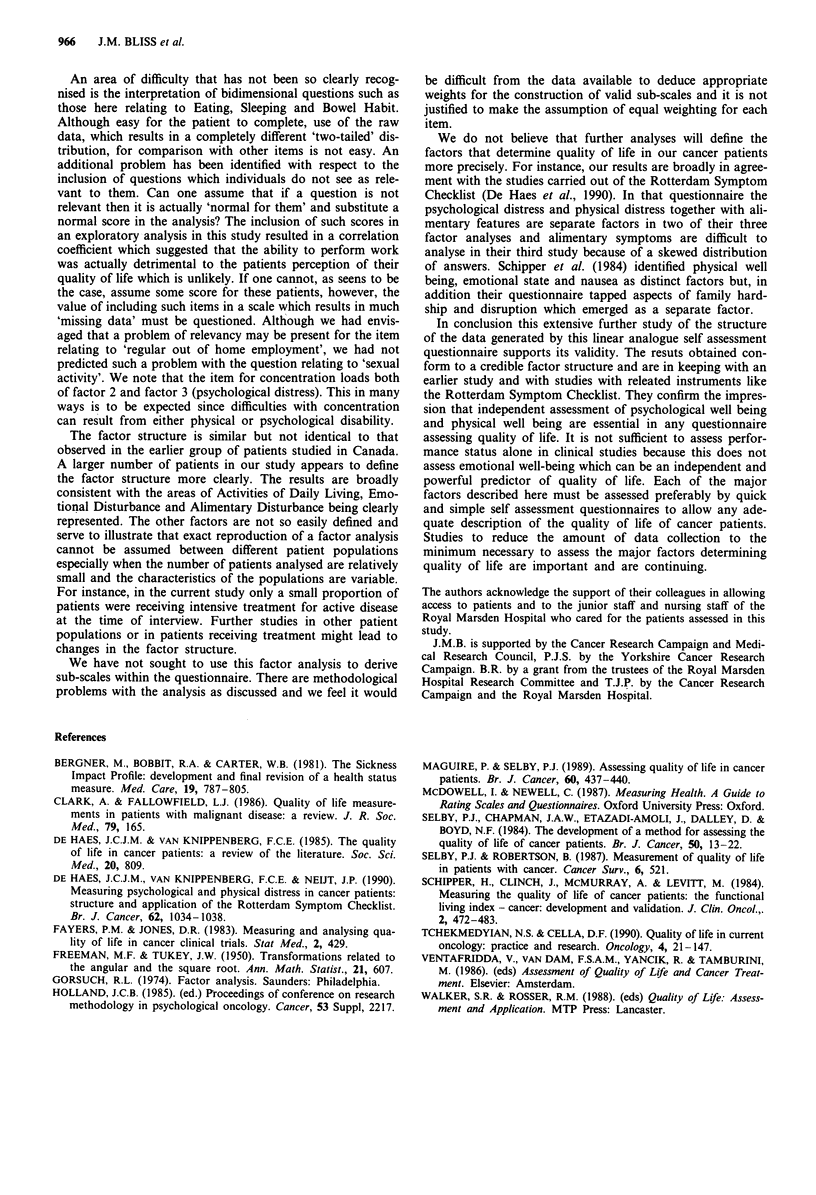# A method for assessing the quality of life of cancer patients: replication of the factor structure.

**DOI:** 10.1038/bjc.1992.201

**Published:** 1992-06

**Authors:** J. M. Bliss, P. J. Selby, B. Robertson, T. J. Powles

**Affiliations:** Section of Epidemiology, Royal Marsden Hospital, Sutton, Surrey, UK.

## Abstract

The psychometric properties of a method of measuring the quality of life of cancer patients based on multiple linear analogue scales have been assessed in a group of 294 patients with breast cancer attending one clinical unit. The method was found to be readily managed by patients although a small number of scales presented difficulties of understanding to patients and difficulties of analysis. The scales distinguished readily between patients of different disease and treatment status. Factor analysis revealed a 5 factor structure which we interpret as relating to physical activities of everyday living, emotional disturbance, alimentary disturbances, appearance and cosmetic problems and a fifth factor which is more difficult to interpret and includes impairment of speech, writing and concentration. We feel the essential factors determining quality of life in cancer patients have been demonstrated in this and our earlier studies and there is now a substantial level of agreement in the factors that have been identified by groups taking quite different approaches. The major factors determining quality of life in cancer patients are now known and should be assessed in clinical research and clinical trials. The method by which they should be assessed is not as yet so clear.


Br. J. Cancer (1992), 65, 961-966 © Macmillan Press Ltd., 1992

### A method for assessing the quality of life of cancer patients: replication of the factor structure

J.M. Bliss[1], P.J. Selby[3], B. Robertson[2] & T.J. Powles[2]

[1]*Section of Epidemiology and* [2]*Section of Medicine, Institute of Cancer Research, Royal Marsden Hospital, Downs Road, Sutton, Surrey SM2 5PT and* [3]*Yorkshire Cancer Research Campaign Institute for Cancer Studies, St James's University Hospital, Beckett Street, Leeds LS9 7TF, UK.*

**Summary** The psychometric properties of a method of measuring the quality of life of cancer patients based on mulitiple linear analogue scales have been assessed in a group of 294 patients with breast cancer attending one clinical unit. The method was found to be readily managed by patients although a small number of scales presented difficulties of understanding to patients and difficulties of analysis. The scales distinguished readily between patients of different disease and treatment status.

Factor analysis revealed a 5 factor structure which we interpret as relating to physical activities of everyday living, emotional disturbance, alimentary disturbances, appearance and cosmetic problems and a fifth factor which is more difficult to interpret and includes impairment of speech, writing and concentration.

We feel the essential factors determining quality of life in cancer patients have been demonstrated in this and our earlier studies and there is now a substantial level of agreement in the factors that have been identified by groups taking quite different approaches. The major factors determining quality of life in cancer patients are now known and should be assessed in clinical research and clinical trials. The method by which they should be assessed is not as yet so clear.

Although there has been over a decade of work towards the development of methods for measuring quality of life in cancer patients, a satisfactory instrument for all purposes fulfilling all recognised requirements has yet to be devised. In a recent review of available instruments, a working party of the Medical Research Council Cancer Therapy Committee concluded 'a multi-dimensional scale which is specific to patients with cancer, meets all the assessment criteria and provides scores which have relevance to clinical judgements remains to be developed' (Maguire & Selby, 1989). Limitations on instruments include the scope of enquiry, design and their interpretation. Only a limited number of instruments have been subjected to rigorous psychometric evaluation for reliability, validity and structure (Clark & Fallowfield, 1986; De Haes & van Knippenberg, 1985; Fayers & Jones, 1983; Holland, 1984; McDowell & Newell, 1987; Selby & Robertson, 1987; Ventafridda *et al.*, 1986; Walker & Rosser, 1988; Tchekmedyian & Cella, 1990).

We earlier reported the development of a method for assessing the quality of life of breast cancer patients based on self assessment by multiple linear analogue scales (Selby *et al.*, 1984). This questionnaire contained 31 items assessed by patients self report including 18 items enquiring about general health problems derived from the Sickness Impact Profile (Bergner *et al.*, 1981) and 13 items enquiring about major problems associated with breast cancer. The method is designed to allow exchange of the items related to breast cancer for those related to other cancer sites. We reported the reliability and validity of the measurement method in the original study which was carried out in Toronto, Canada. The questionnaire performed well in reliability and validty studies and achieved standards which we felt were acceptable for an instrument used in a research setting (Selby *et al.*, 1984).

In evaluation of the performance of a questionnaire of this kind, an important test is the examination of the correlations between individual questions. The techniques of Factor Analysis (Gorsuch, 1974) are used to deduce common factors to which the individual items are correlated and the results of such analysis are known as the factor structure of the data. In the data collected in the Canadian study, in a group of 96 patients, a 5 factor structure was obtained from the questionnaire. Factors were identifed relating to Activities of Every Day Living, Symptoms, Emotional Well-being, Alimentary Well-being and Appearance/Attractiveness.

Work with this method had proceeded in several directions. We have developed alternative 'modules' for alternative cancer sites and subjected these to psychometric evaluation and both this instrument and others developed from it have been used in a number of different settings. Our main intention has been however, to complete the evaluation of the psychometric properties of the original questionnaire and to work towards a reduced instrument that would retain most of the information obtained by asking the 31 questions, but that would be easier and quicker to complete and thus be available for widescale use in clinical research and routine clinical practice. In order to further evaluate the performance of the instrument, we have studied an independent large group of breast cancer patients and we report here a re-evaluation of the factor structure of the questionnaire. Demonstrating stability of the factor structure across different patient populations is an essential element in the adequate evaluation of a measurement method for quality of life studies. Our further studies allow comparison of the factor structure of our method to that of similar instruments developed in other centres. Work concerned with reducing the questionnaire to a shorter instrument with be presented separately.

### Methods

### *Patient population*

The questionnaire was given, on a single occasion, to patients attending the Breast Unit, Royal Marsden Hospital, Sutton over a 15 month period. We attempted to include all eligible patients seen in the clinic. Eligibility was such that the patient must

(a) have a definite diagnosis of breast cancer;
(b) be aware of the diagnosis:
(c) have a fluent understanding of the English language;
(d) be aged <71 years.

Patients with an obvious confusional or psychotic state were

Correspondence: P.J. Selby, YCRC Institute for Cancer Studies, St James's University Hospital, Beckett Street, Leeds LS9 7TF, UK.
Received 2 December 1991; and in revised form 4 March 1992.

excluded as were those with mental impairment and those physically unable to complete the questionnaire without assistance.

The location and treatment status of the patients are given in Tables I and II.

*Structure and questionnaire*

An instrument similar to that used in Canada which included one additional scale relating to sexual activity, was drawn up. A separate five item scale based on the dimensions which had been identified in the Canadian factor analysis, that is Activities of Daily Living, Symptoms, Emotional Well Being, Alimentary Well Being and Appearance/Attractiveness was also included together with a single Uniscale relating to overall quality of life. The scales were all included together in a printed booklet. Three items in the 31 item questionnaire were bipolar (Sleep, Eating and Bowel Habit) and for each of these the patient was asked to complete just one of two linear analogue scales depending on the direction of the morbidity experienced. In practice therefore the questionnaire comprised the 28 individual items listed in Table III, five items derived from the previous factor analysis and an overall QL score. The results reported here evaluate only data for the 28 individual items.

The patients were asked to score how each aspect of their quality of life had been affected by their disease or treatment during the previous 24 h. An initial example was included in the booklet and was shown to the patient to help explain the method of completion. All patients were seen, the questionnaire explained and data collected by one trained interviewer (B.R.). A consecutive series of patients was studied, selected only according to the criteria already stated. Patients attending the hospital both as out-patients and as in-patients were included and after explanation of the procedure, patients were left alone, where possible, to complete the questionnaire. The interviewer returned at a predetermined time and on receipt of the completed questionnaire the items were checked and the patient was asked to complete any items which she had missed. Detailed demographic and clinical data were also collected.

All scales were represented as linear analogue scales (10 cm) (LASA) and for each item a score between 0–100 was calculated, scores of 0 indicating normality or absence of a symptom and those towards 100 indicating severe morbidity or presence of a symptom. Examples of scales are shown in Figure 1. When considering the proportion of patients experiencing morbidity a score of less than 5, i.e. a mark recorded within the lowest 5 mm of the scale, was classed as a normal score. The cut-off for normal scores was taken from the Canadian study and was an arbitrary choice in the earlier study. Missing data for individual items were excluded on a pair-wise basis. One hundred and seventy-four patients had one or more items of missing data (usually the item referring to regular work) but only three patients had more than five missing data items.

Even though bidirectional data had been collected for the items relating to increased and decreased frequencies of sleeping, eating and bowel habit, the data were combined into a simple measure of disturbance since this enabled the data to be represented in a form which could be easily compared with data from the other item on the questionnaire.

*Statistical procedures*

All data was entered into a database using the COMPACT computer software system.

Scores for each of the LASA scales formed a unimodal

**Table III** Ability of LASA scores to distinguish between patient groups

| | *Hospital status* | *Disease status* | *Treatment status* |
|---|---|---|---|
| Uniscale | *** | *** | *** |
| Mobility around home, town or country | *** | *** | *** |
| Social life outside the family | *** | *** | *** |
| Housework | *** | *** | *** |
| Physical activity | *** | *** | ** |
| Recreation, pastimes or hobbies | ** | *** | *** |
| Hair loss | * | *** | *** |
| Fatigue | 0.01 | *** | *** |
| Regular out of home employment | ** | ** | *** |
| Level of anxiety | ** | ** | *** |
| Sexual activity | ** | ** | *** |
| Depression | *** | ** | * |
| Easting disturbance | * | *** | ** |
| Breathing | 0.05 | *** | 0.06 |
| Patin | * | ** | ** |
| Self Care (washing, dressing) | ** | ** | 0.08 |
| Concentration | * | * | ** |
| Appearance of your body | * | * | ** |
| Bowel disturbance | 0.5 | 0.01 | ** |
| Attractiveness to the opposite sex | 0.02 | ** | 0.04 |
| Anger | 0.07 | 0.02 | * |
| Sleep disturbance | 0.06 | 0.02 | * |
| Nausea | 0.03 | 0.03 | 0.01 |
| Speech | 0.02 | 0.02 | 0.04 |
| Vomiting | 0.05 | 0.05 | NS |
| Family relationships | NS | 0.04 | 0.03 |
| Writing | 0.04 | NS | NS |
| Sore mouth | NS | NS | NS |
| Information | NS | NS | NS |

***$P < 0.0001$, **$0.0001 \leqslant P < 0.001$. *$0.001 \leqslant P < 0.01$. p value given $0.01 \leqslant P < 0.1$. NS $P \geqslant 0.1$.

**Table I** Hospital status by status of disease at the time of completion of the questionnaire

| | *Hospital status* | | | | |
|---|---|---|---|---|---|
| *Status of disease* | *Outpatient* | *Day case* | *Recent admission* | *Inpatient* | *Total* |
| Local/regional disease | 14 | 2 | 2 | 8 | 26 (9%) |
| Disease free | 114 | 4 | 0 | 1 | 119 (40%) |
| Distant metastases | 61 | 19 | 27 | 41 | 149 (51%) |
| Total | 189 (64%) | 25 (9%) | 29 (10%) | 51 (17%) | 294 |

**Table II** Current status of treatment

| | *No systemic treatment* | *Chemotherapy* | *Hormonal* | *Chemotherapy and hormonal* |
|---|---|---|---|---|
| No treatment | 145 | 39 | 62 | 4 |
| Radiotherapy | 8 | 5 | 2 | 0 |
| Other treatment | 6 | 9 | 4 | 2 |
| Radiotherapy and other treatment | 1 | 3 | 4 | 0 |

PLEASE SCORE HOW YOU FEEL EACH OF THESE ASPECTS OF YOUR LIFE WAS AFFECTED BY THE STATE OF YOUR HEALTH, DURING TODAY. (24 H).

Physical activity

| | | |
|---|---|---|
| completely unable to move my body | ________________ | normal physical activity for me |

Recreation, pastimes or hobbies

| | | |
|---|---|---|
| completely unable to do them because of the state of my health | ________________ | normal leisure time activities for me |

**Figure 1** Examples of linear analogue scales used in this study.

distribution highly skewed towards the end of the scale representing lack of morbidity. Various transformations were investigated with the aim of normalising the distribution of the data. The modified arc sin transformation proposed by Freeman and Tukey (1950) was found to be the most useful. Analyses were performed with and without transformation and the results were very similar. Results presented here are based on the untransformed data.

The Kruskal-Wallis non parametric analysis of variance or the Mann-Whitney U test were used, depending on the number of levels of the status variable, to test the ability of each item to distinguish between patient groups. A large number of individual comparisons are made in this type of analysis hence absolute $P$-values should be interpreted accordingly and the possibility that some apparent associations may be purely artefactual must be acknowledged. Factor analysis was performed using the SPSSX statistical software package. The suitability of the factor analysis model was tested using various standard measures and the stability of the factor structure was assessed by repeating the analyses using different combinations of extraction and rotational methods. The factor structure identified was found to be reasonably stable across different procedures. The results presented here have used methods of principal components analysis for factor extraction and varimax rotation. This rotational procedure attempts to minimise the number of items that have high loadings on a factor with the aim of enhancing interpretability. Missing data were excluded on a pair-wise basis. The figures shown in the tables are the factor loadings which can be interpreted as the correlation coefficients between the individual items and the chosen factors. All factor loadings with magnitude $>0.4$ are shown, the figures in brackets representing second or smaller factor loading for an item.

### Results

The questionnaire was given to 294 patients. A further seven patients interviewed were subsequently found not to satisfy the eligibility criteria. The study patients represented the broad spectrum of those attending the Breast Clinic and range from out-patients to those admitted for staging and in-patients, all irrespective of whether they were currently receiving treatment.

### *Demographic and clinical details*

The patients were aged between 23 and 70 years with a median age of 55 years. Two hundred and eight patients were married or cohabiting, 29 were single, 19 separated or divorced and 38 widowed. Two hundred and forty-four patients reported having household companions. One hundred and thirty-four patients had regular out of home employment however some of these were unable to work due to the condition of their health.

Table I shows a breakdown of hospital status by status of disease at the time of completion of the questionnaire. One hundred and nineteen (40%) of the patients in our study were apparently free of disease and attending routine follow up and this is a reflection of the patient population seen in the clinic. Table II gives details of current treatment. One hundred and forty-nine (51%) of the patients were currently receiving treatment of some kind, a total of 62 were receiving chemotherapy, 78 hormonal therapy, 23 radiotherapy, 29 some other therapy, with some patients receiving combined treatment schedules. During the previous 24 h only four (6%) of the 62 chemotherapy patients, and six (26%) of the 23 radiotherapy patients had actually received treatment. Treatment had been received during the previous 24 h however, by 58 (75%) of the 78 patients receiving hormonal therapy and 22 (79%) of the 28 patients who were scheduled to receive some other type of treatment.

### *LASA scores*

The data obtained from the questionnaire was of a high standard of completeness. Twenty-one (75%) of the 28 individual item scales, and the Uniscale had missing data for less than six (2%) patients.

For three items, however, more than 5% of patients did not complete the linear analogue scale, these were for the questions relating to attractiveness to the opposite sex (6%), sexual activity (14%) and regular out of home employment (52%). Clearly patients who did not usually work would not consider a question relating to regular out of home employment relevant to them and to allow for this a box had been included on the questionnaire to be completed by such patients. One hundred and fourteen (75%) of the 153 who did not simply complete the linear analogue scale ticked the box. Eleven (7%) ticked the box and marked the line and for eight of these patients ticking the box was appropriate for their stated occupation (i.e. retired/housewife/unemployed), two worked 'at home' and one was disabled. Twenty-eight (18%) left the question blank, 24 of whom had a stated occupation of retired etc. Ony four patients therefore who were in regular out of home employment failed to answer the question. Thirteen patients completed the line and did not tick the box even though they had not reported that they were employed.

A similar problem of applicability to an individual was encountered with the question relating to sexual activity, since several patients wrote a comment to that effect, although this had not been foreseen. It is an over simplification to assume that patients who did not complete this question did so because they did not perceive themselves as experiencing any sexual activity in their 'normal life' and therefore that the question was not applicable to them. However, only seven of the 40 women who did not complete this question were married, the rest being single (nine), divorced (six) or widowed (18).

It appeared therefore that, following instruction, patients had found the questionnaire easy to complete. The sparsity of missing data indicating that, with the exception of the two individual problems already discussed, problems of interpretability of questions had not been experienced. The questionnaire took between 5 and 10 min to complete.

For every item, scores were clustered around the lowest end of the range. The percentage of patients reporting 'normal' (less than 5) scores varying from 28% for fatigue and 29% for anxiety to 93% for vomiting and 93% for speech. For six items (sore mouth, vomiting, self care, speech, writing, information) less than 20% of patients reported 'non-normal' values. From the clinical information it can be seen that the proportion of patients currently receiving treatment which would be likely to cause toxic side effects is small hence it is not surprising that only a small proportion reported such morbidity.

One hundred and thirty-six patients reported 'normal' values for the overall quality of life Uniscale. Only 24 patients, however, scored 'normal' values for each of the 28 items, none of these patients reporting a 'non-normal' Uniscale value. All items were found to be significantly correlated with at least one other item.

*Ability of scales to distinguish between patient groups*

Four classifications of the data, hospital status (out-patient, day case, recent admission, in-patient), present disease status (Local/Regional Disease, Disease Free, Distant Metastases), current treatment status ('on treatment', 'not on treatment') and age ($<55$ years, $\geqslant 55$ years) were used to investigate the ability of the item scores to discriminate between patient groups. If such an ability exists this should increase our confidence in the validity of the measurement recorded.

Table III shows the discriminative ability of the items in relation to hospital, disease and treatment status. No items were found to be strongly associated with age. In each case, any association observed indicated more severe morbidity for the more affected patient group, for example the association between hair loss and treatment status indicates higher levels of morbidity for those patients currently 'on treatment'. Much of the variation with disease status occurs between patients with and without metastatic disease. The inability of some variables to discriminate between patient groups is not surprising given that only a small proportion of patients reported 'non-normal' scores for these items.

*Factor analysis*

Factor analysis is a technique by which the degree to which scores on individual items are correlated to those of other items can be assessed. The aim of the technique is to examine the inter-correlations between each of the individual items and to thereby identify groups of items, the so called factors, within which the scores are most highly correlated statistically. The factor loadings obtained being a measure of the correlation between the individual items and the chosen factor. If the LASA instrument is to be considered of any pratical value, however, the factors selected must be clinically plausible and interpretable.

Considering firstly an analysis which includes all of the 28 individual items, five factors appear to represent the inter-correlations shown in Table IV and this representation of the data explains 59% of the underlying variance. Only one item, that of attractiveness to the opposite sex, does not have a factor loading greater or equal to 0.5. This analysis was repeated excluding the 24 patients who reported 'normal' scores for each of the 28 items and a very similar model was obtained. Exclusion of the item relating to regular out of home employment also did not affect the factor structure and very similar factor loadings were obtained.

Interpreting these factors, Factor 1 appears to represent a dimension, which could be summarised as 'Physical Activities of Daily Living'. Two symptoms, Pain and Fatigue, which may be said to be most closely associated with ones ability to perform normal physical activities are included here in this factor. The association between Pain and Bowel Disturbance which has a possible explanation due to the constipating effects of analgesics should also be noted. It is interesting that the item relating to Attractiveness to the Opposite Sex is included in Factor 1. Factor 2 reveals the expected associations between the different dimensions of emotional disturbance, impairment in family relationships along with the well recognised association in breast cancer with ones perception of appearance of your body. Sleep Disturbance is associated with this emotional factor as well as to the factor relating to activities of daily living. Both Satisfaction with Information given and Concentration are also significantly associated with this dimension as may be expected.

Factor 3 is more difficult to explain but perhaps in some way represents interference of symptoms with minor/specific physical activities e.g lung metastases which result in difficulty in breathing or lymphoedema which may result in difficulty in writing.

Factor 4 appears to represent the Alimentary Disturbances associated with breast cancer and its treatment.

Factor 5 is again not easy to explain but many in some sense represent the cosmetic difficulties associated with treatment. It should be noted that the items relating to attractiveness or appearance do not load heavily on this factor.

The analysis was repeated omiting the six items for which less than 20% of patients reported 'non-normal' values and which therefore may be considered as relatively uninformative in this patient population. The contribution of these items can also be questioned statistically due to the extreme skewness of the distribution of the data.

Table V shows the four factors selected in this analysis. This representation explains 60% of the underlying variance between items. Factor 1 again describes 'Physical Activities of Daily Living', factor 2 now appearing to represent problems associated with the symptoms of the disease. Factor 3 now describes emotional disturbance. Factor 4 is not immediately easy to interpret.

**Table IV** Correlation between items: results of factor analysis – 294 patients, all 28 items

| | *Factor 1* | *Factor 2* | *Factor 3* | *Factor 4* | *Factor 5* |
|---|---|---|---|---|---|
| Housework | 0.76 | | | | |
| Mobility around home, town, country | 0.72 | | | | |
| Regular out of home employment | 0.67 | | | | |
| Physical activity | 0.66 | | | | |
| Social life outside the family | 0.65 | | | | |
| Recreation pastimes or hobbies | 0.63 | | | | |
| Fatigue | 0.62 | | | | |
| Sexual activity | 0.62 | | | | |
| Eating disturbance | 0.60 | | | | |
| Bowel disturbance | 0.58 | | | | |
| Pain | 0.51 | | | | |
| Sleeping disturbance | 0.50 | (0.46) | | | |
| Attractiveness to the opposite sex | 0.43 | | | | |
| Depression | | 0.73 | | | |
| Level of anxiety | | 0.69 | | | |
| Appearance of your body | | 0.68 | | | |
| Anger | | 0.68 | | | |
| Family relationships | | 0.64 | | | |
| Speech | | | 0.72 | | |
| Writing | | | 0.61 | | |
| Breathing | | | 0.60 | | |
| Information | | (0.40) | 0.55 | | |
| Concentration | | (0.49) | 0.50 | | |
| Vomiting | | | | 0.87 | |
| Nausea | | | | 0.84 | |
| Sore mouth | | | | 0.52 | |
| Self care (washing, dressing) | | | | | 0.67 |
| Hair loss | | | | | 0.55 |

Note: Figures shown are rotated factor loadings. Figures in parentheses indicating secondary factor loadings $>0.4$.

**Table V** Correlation between items: results of factor analysis – all patients, exclusion of items with <20% non-normal values

| | *Factor 1* | *Factor 2* | *Factor 3* | *Factor 4* |
|---|---|---|---|---|
| Recreation, pastimes or hobbies | 0.75 | | | |
| Physical activity | 0.72 | | | (0.41) |
| Housework | 0.69 | | | |
| Mobility | 0.68 | | | (0.42) |
| Social life outside the family | 0.62 | | | |
| Bowel disturbance | 0.58 | (0.45) | | |
| Sexual activity | 0.57 | | | |
| Hair loss | 0.55 | | | |
| Regular out of home employment | 0.52 | | | |
| Eating disturbance | | 0.73 | | |
| Sleeping disturbance | | 0.68 | | |
| Fatigue | | 0.62 | | |
| Nausea | | 0.57 | | |
| Pain | | 0.46 | | |
| Anger | | | 0.78 | |
| Depression | | | 0.73 | |
| Appearance of your body | | | 0.67 | |
| Anxiety | | | 0.64 | |
| Concentration | | (0.41) | 0.42 | |
| Breathing | | | | 0.68 |
| Family relationships | | | (0.51) | 0.60 |
| Attractiveness to the opposite sex | | | | 0.52 |

If one excludes the patients who reported a 'normal' score in response to the assessment of overall quality of life, by the Uniscale, a five factor model is chosen to describe the intercorrelations which exist and is shown in Table VI. This model explains 56% of the underlying variance between items. A factor structure very similar to that found when considering all patients is observed.

Remarkable stability in the factor structure is observed across the analyses. With the exception of the factor depicted as 'cosmetic' effect and to a lesser extent that of physical impairment due to symptoms, the observed associations are clearly plausible and easily interpretable.

Further analyses performed on patient subsets were not felt to significantly alter the conclusions on the structure of the data already obtained.

### *Comparison of the factor analysis with the Canadian study*

In the Canadian study a factor analysis was performed to assess validity using a group of 96 breast cancer patients with recurrent disease. Fifty-two (54%) were currently receiving chemotherapy and the mean age of this sample was 57 years. The results of the analysis are shown in Table VII. All factor loadings greater than 0.3 are included. In our analyses, with a much larger patient group and more heterogeneity, we include all factor loadings greater than 0.4. Very few items in our current analysis have a factor loading less than 0.5.

In the Canadian analysis items relating to Sore Mouth, Dysuria (which we did not measure), Speech and Self Care were not included due to the narrow distribution of the data. The item relating to regular out of home employment was also excluded due to the problem of missing data. We have presented analyses with and without these items and have shown that although their inclusion suggests an additional factor, the main structure is consistent across the analyses.

The factor structure appears to be more clearly defined in the current study than in the Canadian study. This may be due to the smaller number of patients included in the Canadian study. However, the results do appear to be broadly consistent. Factors 1 and 2 from the Canadian study appearing to describe Activities of Daily Living, Factor 3 Emotional Disturbance, Factor 4 Alimentary Disturbance. Factor 5 is less easy to compare. A clear appearance/attractiveness factor does not emerge in our current study.

**Table VI** Correlation between items: results of factor analysis – exluding 136 patients who reported a 'normal' overall quality of life, Uniscale, score

| | *Factor 1* | *Factor 2* | *Factor 3* | *Factor 4* | *Factor 5* |
|---|---|---|---|---|---|
| Physical activity | 0.76 | | | | |
| Housework | 0.75 | | | | |
| Mobility | 0.73 | | | | |
| Recreation, pastimes or hobbies | 0.73 | | | | |
| Social life outside the family | 0.70 | | | | |
| Bowel disturbance | 0.56 | | | | |
| Sexual activity | 0.56 | | | | |
| Attractiveness to the opposite sex | 0.55 | | | | |
| Regular out of home employment | 0.43 | | | | |
| Pain | 0.41 | | | | |
| Fatigue | 0.41 | | | | |
| Depression | | 0.74 | | | |
| Level of anxiety | | 0.73 | | | |
| Appearance of your body | | 0.68 | | | |
| Anger | | 0.64 | | | |
| Family relationships | | 0.64 | | | |
| Sleep disturbance | | 0.48 | | | |
| Breathing | | | 0.70 | | |
| Speech | | | 0.70 | | |
| Writing | | | 0.61 | | |
| Information | | | 0.55 | | |
| Concentration | | (0.50) | 0.53 | | |
| Vomiting | | | | 0.85 | |
| Nausea | | | | 0.83 | |
| Eating disturbance | (0.50) | | | 0.50 | |
| Self care (washing, dressing) | | | | | 0.64 |
| Hair loss | | | | | 0.63 |
| Sore mouth | | | | | 0.46 |

**Table VII** Results of Canadian study (from Selby *et al.*, 1984)

| | *Factor 1* | *Factor 2* | *Factor 3* | *Factor 4* | *Factor 5* |
|---|---|---|---|---|---|
| Housework | 0.89 | | | | |
| Recreation, pastimes, hobbies | 0.85 | | | | |
| Social life | 0.71 | | | | |
| Mobility | 0.49 | | | | |
| Fatigue | 0.46 | | | | |
| Writing | 0.44 | | | | |
| Pain | | 0.60 | | | |
| Physical activity | 0.42 | 0.57 | | | |
| Bowel habit | | 0.54 | | | |
| Breathing | | 0.42 | | | |
| Depression | | | 0.80 | | |
| Anger | | | 0.77 | | |
| Anxiety | | | 0.68 | | |
| Appearance | | | 0.40 | | |
| Concentration | 0.40 | | 0.40 | | |
| Nausea | | | | 0.86 | |
| Vomiting | | | | 0.85 | |
| Eating | | | | 0.38 | |
| Attractiveness | | | | | 0.64 |
| Family relations | | | 0.38 | | 0.63 |
| Hair loss | | | | | 0.35 |

### Discussion

This study confirms the ability of this instrument to discriminate between patients of different circumstances, different disease and treatment status. This analysis has further illustrated the limitations of linear analogue scales in that the skewed data obtained are difficult to manipulate statistically. Some areas of morbidity were only seen to cause concern to a small proportion of patients. However we have shown that inclusion of such items in a factor analysis does not affect the main factors identified.

An area of difficulty that has not been so clearly recognised is the interpretation of bidimensional questions such as those here relating to Eating, Sleeping and Bowel Habit. Although easy for the patient to complete, use of the raw data, which results in a completely different 'two-tailed' distribution, for comparison with other items is not easy. An additional problem has been identified with respect to the inclusion of questions which individuals do not see as relevant to them. Can one assume that if a question is not relevant then it is actually 'normal for them' and substitute a normal score in the analysis? The inclusion of such scores in an exploratory analysis in this study resulted in a correlation coefficient which suggested that the ability to perform work was actually detrimental to the patients perception of their quality of life which is unlikely. If one cannot, as seens to be the case, assume some score for these patients, however, the value of including such items in a scale which results in much 'missing data' must be questioned. Although we had envisaged that a problem of relevancy may be present for the item relating to 'regular out of home employment', we had not predicted such a problem with the question relating to 'sexual activity'. We note that the item for concentration loads both of factor 2 and factor 3 (psychological distress). This in many ways is to be expected since difficulties with concentration can result from either physical or psychological disability.

The factor structure is similar but not identical to that observed in the earlier group of patients studied in Canada. A larger number of patients in our study appears to define the factor structure more clearly. The results are broadly consistent with the areas of Activities of Daily Living, Emotional Disturbance and Alimentary Disturbance being clearly represented. The other factors are not so easily defined and serve to illustrate that exact reproduction of a factor analysis cannot be assumed between different patient populations especially when the number of patients analysed are relatively small and the characteristics of the populations are variable. For instance, in the current study only a small proportion of patients were receiving intensive treatment for active disease at the time of interview. Further studies in other patient populations or in patients receiving treatment might lead to changes in the factor structure.

We have not sought to use this factor analysis to derive sub-scales within the questionnaire. There are methodological problems with the analysis as discussed and we feel it would be difficult from the data available to deduce appropriate weights for the construction of valid sub-scales and it is not justified to make the assumption of equal weighting for each item.

We do not believe that further analyses will define the factors that determine quality of life in our cancer patients more precisely. For instance, our results are broadly in agreement with the studies carried out of the Rotterdam Symptom Checklist (De Haes *et al.*, 1990). In that questionnaire the psychological distress and physical distress together with alimentary features are separate factors in two of their three factor analyses and alimentary symptoms are difficult to analyse in their third study because of a skewed distribution of answers. Schipper *et al.* (1984) identified physical well being, emotional state and nausea as distinct factors but, in addition their questionnaire tapped aspects of family hardship and disruption which emerged as a separate factor.

In conclusion this extensive further study of the structure of the data generated by this linear analogue self assessment questionnaire supports its validity. The resuts obtained conform to a credible factor structure and are in keeping with an earlier study and with studies with releated instruments like the Rotterdam Symptom Checklist. They confirm the impression that independent assessment of psychological well being and physical well being are essential in any questionnaire assessing quality of life. It is not sufficient to assess performance status alone in clinical studies because this does not assess emotional well-being which can be an independent and powerful predictor of quality of life. Each of the major factors described here must be assessed preferably by quick and simple self assessment questionnaires to allow any adequate description of the quality of life of cancer patients. Studies to reduce the amount of data collection to the minimum necessary to assess the major factors determining quality of life are important and are continuing.

The authors acknowledge the support of their colleagues in allowing access to patients and to the junior staff and nursing staff of the Royal Marsden Hospital who cared for the patients assessed in this study.

J.M.B. is supported by the Cancer Research Campaign and Medical Research Council, P.J.S. by the Yorkshire Cancer Research Campaign. B.R. by a grant from the trustees of the Royal Marsden Hospital Research Committee and T.J.P. by the Cancer Research Campaign and the Royal Marsden Hospital.

### References

BERGNER, M., BOBBIT, R.A. & CARTER, W.B. (1981). The Sickness Impact Profile: development and final revision of a health status measure. *Med. Care*, **19**, 787-805.

CLARK, A. & FALLOWFIELD, L.J. (1986). Quality of life measurements in patients with malignant disease: a review. *J. R. Soc. Med.*, **79**, 165.

DE HAES, J.C.J.M. & VAN KNIPPENBERG, F.C.E. (1985). The quality of life in cancer patients: a review of the literature. *Soc. Sci. Med.*, **20**, 809.

DE HAES, J.C.J.M., VAN KNIPPENBERG, F.C.E. & NEIJT, J.P. (1990). Measuring psychological and physical distress in cancer patients: structure and application of the Rotterdam Symptom Checklist. *Br. J. Cancer*, **62**, 1034-1038.

FAYERS, P.M. & JONES, D.R. (1983). Measuring and analysing quality of life in cancer clinical trials. *Stat Med.*, **2**, 429.

FREEMAN, M.F. & TUKEY, J.W. (1950). Transformations related to the angular and the square root. *Ann. Math. Statist.*, **21**, 607.

GORSUCH, R.L. (1974). Factor analysis. Saunders: Philadelphia.

HOLLAND, J.C.B. (1985). (ed.) Proceedings of conference on research methodology in psychological oncology. *Cancer*, **53** Suppl, 2217.

MAGUIRE, P. & SELBY, P.J. (1989). Assessing quality of life in cancer patients. *Br. J. Cancer*, **60**, 437-440.

MCDOWELL, I. & NEWELL, C. (1987). *Measuring Health. A Guide to Rating Scales and Questionnaires.* Oxford University Press: Oxford.

SELBY, P.J., CHAPMAN, J.A.W., ETAZADI-AMOLI, J., DALLEY, D. & BOYD, N.F. (1984). The development of a method for assessing the quality of life of cancer patients. *Br. J. Cancer*, **50**, 13-22.

SELBY, P.J. & ROBERTSON, B. (1987). Measurement of quality of life in patients with cancer. *Cancer Surv.*, **6**, 521.

SCHIPPER, H., CLINCH, J., MCMURRAY, A. & LEVITT, M. (1984). Measuring the quality of life of cancer patients: the functional living index - cancer: development and validation. *J. Clin. Oncol.*,. **2**, 472-483.

TCHEKMEDYIAN, N.S. & CELLA, D.F. (1990). Quality of life in current oncology: practice and research. *Oncology*, **4**, 21-147.

VENTAFRIDDA, V., VAN DAM, F.S.A.M., YANCIK, R. & TAMBURINI, M. (1986). (eds) *Assessment of Quality of Life and Cancer Treatment.* Elsevier: Amsterdam.

WALKER, S.R. & ROSSER, R.M. (1988). (eds) *Quality of Life: Assessment and Application.* MTP Press: Lancaster.